# A multimodal radiomic machine learning approach to predict the LCK expression and clinical prognosis in high-grade serous ovarian cancer

**DOI:** 10.1038/s41598-023-43543-7

**Published:** 2023-09-29

**Authors:** Feng Zhan, Lidan He, Yuanlin Yu, Qian Chen, Yina Guo, Lili Wang

**Affiliations:** 1 School of Electronic Information Engineering, Taiyuan University of Science and Technology, Taiyuan, Shanxi People’s Republic of China; 2 College of Engineering, Fujian Jiangxia University, Fuzhou, Fujian People’s Republic of China; 3 Department of Obstetrics and Gynecology, The First Affiliated Hospital of Fujian Medical University, Fuzhou, Fujian People’s Republic of China; 4 Department of Medical Imaging, The First Affiliated Hospital of Fujian Medical University, Fuzhou, Fujian People’s Republic of China; 5 Department of Radiology, Fujian Medical University Union Hospital, Fuzhou, Fujian People’s Republic of China

**Keywords:** Ovarian cancer, Tumour biomarkers, Biomarkers

## Abstract

We developed and validated a multimodal radiomic machine learning approach to noninvasively predict the expression of lymphocyte cell-specific protein-tyrosine kinase (LCK) expression and clinical prognosis of patients with high-grade serous ovarian cancer (HGSOC). We analyzed gene enrichment using 343 HGSOC cases extracted from The Cancer Genome Atlas. The corresponding biomedical computed tomography images accessed from The Cancer Imaging Archive were used to construct the radiomic signature (Radscore). A radiomic nomogram was built by combining the Radscore and clinical and genetic information based on multimodal analysis. We compared the model performances and clinical practicability via area under the curve (AUC), Kaplan–Meier survival, and decision curve analyses. LCK mRNA expression was associated with the prognosis of HGSOC patients, serving as a significant prognostic marker of the immune response and immune cells infiltration. Six radiomic characteristics were chosen to predict the expression of LCK and overall survival (OS) in HGSOC patients. The logistic regression (LR) radiomic model exhibited slightly better predictive abilities than the support vector machine model, as assessed by comparing combined results. The performance of the LR radiomic model for predicting the level of LCK expression with five-fold cross-validation achieved AUCs of 0.879 and 0.834, respectively, in the training and validation sets. Decision curve analysis at 60 months demonstrated the high clinical utility of our model within thresholds of 0.25 and 0.7. The radiomic nomograms were robust and displayed effective calibration. Abnormally high expression of LCK in HGSOC patients is significantly correlated with the tumor immune microenvironment and can be used as an essential indicator for predicting the prognosis of HGSOC. The multimodal radiomic machine learning approach can capture the heterogeneity of HGSOC, noninvasively predict the expression of LCK, and replace LCK for predictive analysis, providing a new idea for predicting the clinical prognosis of HGSOC and formulating a personalized treatment plan.

## Introduction

Ovarian, endometrial, and cervical cancers are the three most prevalent gynecological malignancies in medical obstetrics and gynecology that endanger women’s health and lives^1–3^. High-grade serous ovarian cancer (HGSOC), the most prevalent histological subtype of ovarian cancer (OC), has the worst prognosis^4, 5^. Despite the remarkable achievements of surgery, chemotherapy, targeted therapy, and immunotherapy, the overall survival (OS) rate of OC remains poor^6^. This is due to the considerable heterogeneity between patients and within tumors, which is linked to adverse clinical outcomes in OC^7^. The clinical requirements of precision medicine cannot be satisfied by traditional prognostic indicators, such as clinicopathological features and serum markers, as well as traditional imaging indicators. Therefore, it is necessary to explore new prognostic markers that can be used to predict individualized precision therapy.

Tumors are comprised of a heterogeneous population of cells with distinct genetic and molecular profiles^8^. The analysis of specific classes and subclasses of the tumor immune microenvironment (TIME) within a patient's tumor can enhance the ability to predict and guide the effectiveness of immunotherapy, as well as identify new therapeutic targets^9^. Lymphocyte cell-specific protein-tyrosine kinase (LCK) is a 56 kDa protein found in lymphocytes, specialized cells of the immune system^10^. Bioinformatic analysis of core immune escape-related genes identifies LCK as a prognostic biomarker capable of modulating the tumor microenvironment (TME)^11^. LCK plays a role in the intracellular signal transduction of lymphocytes through phosphorylation and may serve as a potential biomarker for distinguishing primary central nervous system lymphoma from glioblastoma multiforme^12^. High expression of LCK in OC can better predict progression-free survival and OS than a cytolytic activity score^13^. Several clinical trials targeting LCK are currently underway due to its prominent role in the regulation of immunity, involving cancers^8, 14^, inflammatory diseases^15^, etc. LCK is a downstream molecule in T-cell receptor signaling pathways in several cancer types. It is positively linked to both T-cell-mediated and B-cell-mediated antitumor immune responses. Although the detection of LCK is important, its invasive nature presents a challenge, and non-invasive methods for predicting the level of LCK expression in OC are currently lacking.

Medical imaging techniques, including ultrasound, computed tomography (CT), and magnetic resonance imaging, are widely used for diagnosis and evaluation OC due to their non-invasive and convenient nature. However, traditional imaging methods are limited to their ability to discern the intra- or inter-tumoral heterogeneity of OC^16^. In recent years, radiomics is a rapidly developing method that enables medical imaging to access mineable high-dimension semantic features; it combines qualitative and/or quantitative imaging data for clinical diagnosis and prognosis and is a non-invasive, dynamic-detection, quantitative approach for tumor characterization^17–19^. Furthermore, radiomics, combined with machine learning, has demonstrated its efficacy in predicting the OS of patients^20, 21^. Previous studies have shown that radiomics can be employed for the early identification, classification, and diagnosis of OC as well as for the evaluations of the tumor microenvironment, lymph node load, residual disease, and tumor heterogeneity^22–24^. However, to the best of our knowledge, no research has examined how well a radiomic model predicts the level of LCK expression and enables non-invasive prognosis in the clinical setting. In this study, we propose a novel diagnostic method to address the abovementioned technical challenges of traditional techniques. Additionally, we employ bioinformatics analysis to investigate the potential molecular mechanisms underlying LCK expression and its interaction with the immunological microenvironment. Moreover, we construct a radiomic model for non-invasive prediction of LCK expression, and investigate its relationship with the clinical prognosis of HGSOC patients. Furthermore, we assess the feasibility of radiomics as a non-invasive approach for predicting the mRNA expression of LCK in HGSOC tissue. Finally, we compare the performance of our predictive radiomic model with established clinical features and prognosis. The development of predictive models for clinical outcomes has the potential to serve as a valuable tool in the clinical environment.

## Materials and methods

### Data retrieval and analysis

**Figure 1:**
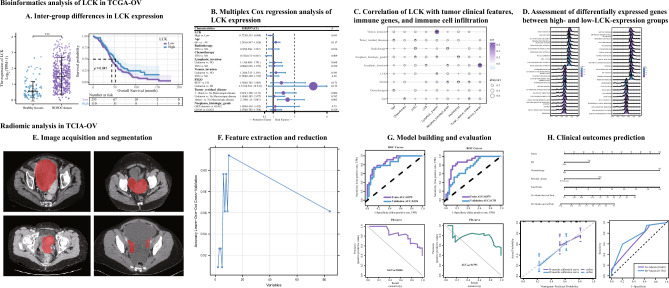
Workflow chart of the study process on HGSOC. *HGSOC* high-grade serous ovarian cancer.

We accessed data for patients with HGSOC from The Cancer Imaging Archive (TCIA) and The Cancer Genome Atlas (TCGA) public repositories^25^. In the TCGA database, we used the Research Network tool (https://portal.gdc.cancer.gov/) to retrieve complete transcriptome sequencing data and clinical information for these patients (such as clinical and follow-up information). Data for available phenotypic variables (age, sex, and OS) were also downloaded from TCGA. Biomedical CT images were accessed from the TCIA website (https://www.cancerimagingarchive.net/)^26^. A workflow chart is summarized in Fig. 1. The TCIA-TCGA public portal’s data usage guideline was followed when using the public datasets.

Our study included 343 HGSOC cases retrieved from TCGA. We excluded cases lacking survival data, those in which the survival time was less than one month, and those in which HGSOC was not the primary solid tumor. The 343 samples were used to identify critical molecules as significant prognostic markers and to carry out enrichment analysis. Our HGSOC CT image dataset included 91 samples from TCIA data. Samples were included if the patient had yet to receive any treatment before the CT examination. Exclusion criteria comprised images with incomplete accompanying clinical information. Among the 91 samples, 57 intersected with the bioinformatics information in TCGA data (used to predict LCK expression by radiomics), and 89 intersected with the clinical information in TCGA data (these were used to explore the association between radiomics and actual prognosis). The gene threshold was selected as 1.322, corresponding with the threshold employed during the bioinformatics analysis. All qualified sample groups were dichotomized into high- and low-LCK-expression groups depending on the cutoff value using the survMisc package in R programming language.

### Bioinformatics analysis of LCK

#### Inter-group differences in LCK expression

HGSOC tissue data were extracted from TCGA, and healthy tissue data for comparative purposes were extracted from The Genotype–Tissue Expression (GTEx) dataset (https://gtexportal.org/home/)^27^. The University of California Santa Cruz Xena (https://xenabrowser.net/datapages/) RNA-sequencing (RNA-seq) dataset was universally transformed with the Toil procedure into transcripts per million format for the TCGA and GTEx^28, 29^. Then, the RNA-seq data were log_2_-transformed for comparison of gene expression between samples.

Changes in the survival rates of various groups were displayed using the Kaplan–Meier (K–M) survival curve. The median survival time was defined as a 50% survival rate. The importance of different survival rates between groups was examined using the log-rank test.

#### Multiplex Cox regression analysis

The Cox proportional hazards model can assess the association between single or multiple factors and survival outcomes^30^. To investigate the variables affecting OS, we performed a correlation coefficient analysis using univariate Cox regression. To determine the relative importance of various contributing factors and whether any particular component could significantly impact the OS, multivariate Cox regression analysis was used. The independent variable was considered as a potential risk factor when the hazard ratio (HR) exceeded 1. When the HR was less than 1, the independent variable was considered as a protective factor.

To determine the impact of high and low LCK expression on the prognosis of patients in various subgroups of each covariate, exploratory subgroup analysis with univariate Cox regression was carried out. A likelihood ratio test was used to evaluate the interaction between LCK and the other covariates.

#### Analysis of the correlation of LCK with tumor clinical features, immune genes, and immune cell infiltration

The Spearman rank correlation coefficient was used to analyze the relationships between LCK and clinical tumor features, immune genes, and immune cell infiltration. Gene expression matrices of HGSOC were uploaded to the cell type identification by estimating relative subsets of known RNA transcripts (CIBERSORTx) database (https://cibersortx.stanford.edu/), which was adopted to calculate immune cell infiltration for each sample^31, 32^.

#### Assessment of differentially expressed genes between high- and low-LCK-expression groups

To explore the molecular mechanisms underlying differential genes between the high- and low-LCK-expression groups, we performed gene set enrichment analysis (GSEA) on the Kyoto Encyclopedia of Genes and Genomes (KEGG) and Hallmark gene sets^33–35^.

### Construction of a multimodal radiomic machine learning model

#### Image interpretation and tumor segmentation

The delineation of the region of interest (ROI) and the volume of interest (VOI) is crucial for quantitative analysis of medical image features. Two radiologists, one with 10 years (reader A) and one with 15 years (reader B) of professional training in gynecological radiography, independently analyzed all images with a 3D slicer software to segment the ROI and VOI of HGSOC^36^. The VOIs of all cases were outlined along the tumor contour by reader A. To evaluate the between-group consistency of ROI and VOI determination, reader B randomly selected data from twenty patients (using the random number table method) to repeat the ROI and VOI determination; irrelevant organs and tissues were excluded as much as possible during these assessments. Both radiologists were blinded to information on clinical factors and HGSOC status. The two radiologists were made aware that all patients had HGSOC because this study did not evaluate the capability of CT to identify HGSOC. They analyzed the CT images from multiple planes (including the axial, coronal, and sagittal planes) to achieve a more precise assessment of HGSOC.

#### Radiomic feature extraction/selection and model construction

To determine radiomic expression patterns, we obtained radiomic features from the HGSOC CT dataset. All DICOM series were converted to three-dimensional pictures, and an abdominal imaging window was added (level 50 and width 400). The SimpleITK image analysis toolbox was used to resample the pictures to isotropic 1 mm^3^ voxels. Radiomics extracts many quantitative features to represent the phenotypic variations among malignancies. PyRadiomics (version 3.0.1) was employed to derive radiomic features from the segmented labels of the HGSOC scans^37^, as executed in Python (version 3.9). We evaluated the radiomic feature model’s functionality using various feature selection methods. The computation of the radiomic feature precisely followed the Image Biomarker Standardization Initiative’s recommendations^38^. The inter-reader reproducibility and reliability of tumor segmentation and radiomic feature extraction were assessed using intraclass correlation coefficients (ICCs). The predictors were sorted before modeling, and the less significant factors were gradually discarded. The objective was to identify a subgroup of predictors that could be used to generate accurate models. For this purpose, the recursive feature elimination algorithm was used to filter out the best subset of radiomic features.

The radiomic models were generated using support vector machines (SVM) and logistic regression (LR). Each linear regression was transformed with the sigmoidal function to obtain output values distributed between 0 and 1. The selected radiomic features were fitted with the LR algorithm to create a binary classification model for forecasting LCK expression.

#### Radiomic model performance assessment

Cross-validation is a commonly used method for constructing validation sets^39, 40^. Cross-validation is an effective method for avoiding overfitting and underfitting, enhancing the model's generalization ability, and is more suitable for small-sample datasets^41, 42^. The performance of radiomic model was evaluated using five-fold cross-validation. To evaluate the performance of the radiomic model, various indices including the area under the curve (AUC), accuracy, sensitivity, specificity, positive predictive value (PPV), negative predictive value (NPV), and Brier score were utilized. Receiver operating characteristic (ROC) and precision-recall (PR) curves were generated to evaluate the radiomic model. The area under the PR curve (PR-AUC) represented the median of the precision computed for each coverage threshold. With the help of the calibration curve and Hosmer–Lemeshow test, our radiomic prediction model was assessed. The clinical utility of the radiomic prediction model was evaluated using decision curve analysis (DCA).

We also examined the potential associations of the LR and SVM radiomic models in predicting the probability of LCK expression. A radiomic signature (Radscore) was defined as the probability of the model output accurately predicting the gene expression level. We used the Wilcoxon test to determine whether the radiomic markers differed between the high- and low-LCK-expression groups. The AUC values of the LR and SVM radiomic models were compared using the Delong test during training and validation within the five-fold cross-validation.

#### Association of radiomic feature with clinical information

The Radscore of the LR radiomic model was combined with clinical data. The cutoff Radscore value determined with the survminer package was 0.254, which was used for classification into high and low Radscore groups.

### Prediction of clinical prognosis using the multimodal radiomic nomogram

The performance of the radiomic nomogram was quantitatively evaluated using the C-index. Briefly, a stepwise regression algorithm screened the clinical variables according to the Akaike information criterion (AIC), which balances model complexity with goodness-of-fit. Clinical and Radscore variables were selected to build a predictive model by choosing the smallest AIC value. We plotted a nomogram of the 36-month and 60-month survival probabilities based on Cox regression.

The ROC curve of the predictive model was used to assess a variable’s predictive ability at various time intervals. The abscissa of the calibration chart showed the actual survival rate, the diagonal axis showed the anticipated probability equal to the actual probability, and the ordinate represented the predicted survival rate. The clinical benefit of the radiomic prediction model was evaluated via DCA.

### Statistical analysis

RStudio (version 4.2) software was used to carry out the statistical analysis. The following R packages were utilized: the irr package for calculating ICC values; the caret package for feature screening; the stats package for radiomic model construction; the ggpubr package for analyzing differences between groups; the survminer package for determining the high- and low-expression of radiomic score indicators; and the survival package for survival analysis of each variable. Statistical significance was set at *p* < 0.05 (*), *p* < 0.01 (**), and *p* < 0.001 (***).

### Ethical approval

This study was performed in line with the principles of the Declaration of Helsinki. Approval was granted by the Ethics Committee of the First Affiliated Hospital of Fujian Medical University (No.: IEC-FOM-013-2.0).

### Consent to participate

Due to the retrospective nature of this study, the requirements for informed consent were waived by the Ethics Committee of the First Affiliated Hospital of Fujian Medical University.

## Results

### Clinical characteristics of LCK expression

Using a cutoff value of 1.32 for LCK expression, we divided the 343 cases of HGSOC from TCGA data into two groups: low LCK expression (*n* = 233) and high LCK expression (*n* = 110). The clinical details of the patients and tumor characteristics are presented in Table 1. Age, radiation, chemotherapy, lymphatic invasion, venous invasion, tumor residual disease, and histologic neoplasm grade were not significantly different between the high- and low-LCK-expression groups (*p* > 0.05). However, the International Federation of the Gynecology and Obstetrics (FIGO) stage differed significantly between the two cohorts (*p* < 0.05).

**Table 1:** Patients and tumor characteristics of the high- and low-LCK-expression groups.

Characteristic	Total (n = 343) No. (%)	Low (n = 233) No. (%)	High (n = 110) No. (%)	*p*
Age (years)				0.743
< 59	178 (52)	119 (51)	59 (54)	
≥ 60	165 (48)	114 (49)	51 (46)	
Radiotherapy				1
No	321 (94)	218 (94)	103 (94)	
Yes	22 (6)	15 (6)	7 (6)	
Chemotherapy				0.793
No	22 (6)	16 (7)	6 (5)	
Yes	321 (94)	217 (93)	104 (95)	
Lymphatic invasion				0.135
No	40 (12)	32 (14)	8 (7)	
Unknown	210 (61)	143 (61)	67 (61)	
Yes	93 (27)	58 (25)	35 (32)	
Venous invasion				0.971
No	32 (9)	22 (9)	10 (9)	
Unknown	251 (73)	171 (73)	80 (73)	
Yes	60 (17)	40 (17)	20 (18)	
FIGO stage				0.014
I/II	19 (6)	8 (3)	11 (10)	
III/IV	321 (94)	224 (96)	97 (88)	
Unknown	3 (1)	1 (0)	2 (2)	
Tumor residual disease				0.572
No. Macroscopic disease	58 (17)	39 (17)	19 (17)	
1–10 mm	162 (47)	111 (48)	51 (46)	
Unknown	36 (10)	21 (9)	15 (14)	
≥ 10 mm	87 (25)	62 (27)	25 (23)	
Neoplasm histologic grade				0.711
G1/G2	42 (12)	31 (13)	11 (10)	
GX/unknown	8 (2)	6 (3)	2 (2)	
G3/G4	293 (85)	196 (84)	97 (88)	

#### Inter-group differences in LCK expression

**Figure 2:**
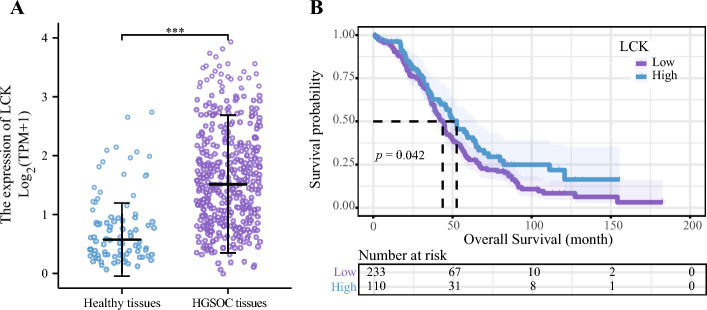
A comparison of the LCK expression level between the normal tissues and ovarian cancer tissues and the comparison of survival data. (**A**) Scatter plot of LCK expression between the healthy and HGSOC tissues. (**B**) Survival chart of LCK and survival probability. *p < 0.05, which is statistically significant; **p < 0.01, which is highly statistically significant; ***p < 0.001, which is very statistically significant. *LCK* lymphocyte cell-specific protein-tyrosine kinase, *HGSOC* high-grade serous ovarian cancer.

There was a noticeably higher LCK expression in HGSOC tissues than in healthy tissues (*p* < 0.01) (Fig. 2A). The mean survival time of the low-LCK-expression group was 43.8 months, whereas that of the high-LCK-expression group was 52.63 months. A high expression of LCK was significantly associated with improved OS, according to the K–M curve (*p* = 0.042) (Fig. 2B).

#### Multiplex Cox regression analysis of LCK expression

**Figure 3:**
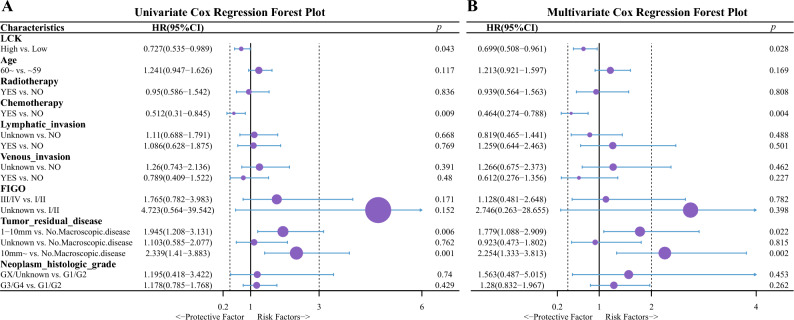
Analysis of the correlation of OS with clinical characteristics using uni- and multi-variate Cox regression. (**A**) Univariate Cox regression analysis. (**B**) Multivariate Cox regression analysis. *LCK* lymphocyte cell-specific protein-tyrosine kinase, *FIGO* international federation of the gynecology and obstetrics, *HR* hazard ratio, *CI* confidence interval.

**Figure 4:**
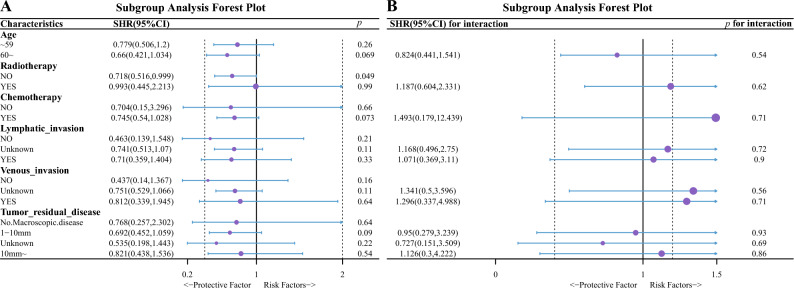
Effect of LCK on patient prognosis in different subgroups utilizing univariate Cox regression analysis. (**A**) The Impact of LCK expression levels on patient prognosis in different subgroups. (**B**) The interaction between LCK and other covariables by likelihood ratio test. Because the sample size for analysis of LCK expression in the Neoplasm_histologic_grade subgroup was too small and the HR values were extreme, the Neoplasm_histologic_grade subgroup was excluded. *SHR* subgroup hazard ratio, *CI* confidence interval.

A high expression of LCK was a statistically significant protective factor for OS in univariate Cox regression analysis (HR = 0.727, 95% CI 0.535–0.989, *p* = 0.043) (Fig. 3A). Moreover, a high expression of LCK was a statistically significant protective factor for OS in multivariate Cox regression analysis (HR = 0.699, 95% CI 0.508–0.961, *p* = 0.028) (Fig. 3B). Elevated LCK expression was a significant protective factor (HR = 0.718, 95% CI 0.516–0.999, *p* = 0.049) for HGSOC in the subgroup that did not receive radiotherapy (Fig. 4A); in contrast, elevated LCK expression was not a protective factor (HR = 0.993, 95% CI 0.445–2.213, *p* = 0.99) for HGSOC in the subgroup that did receive radiotherapy (Fig. 4B). There was no significant interaction in terms of LCK between patients with and without radiotherapy and association between LCK and the OS of patients.

#### Analysis of the correlation of LCK with tumor clinical features, immune genes, and immune cell infiltration

**Figure 5:**
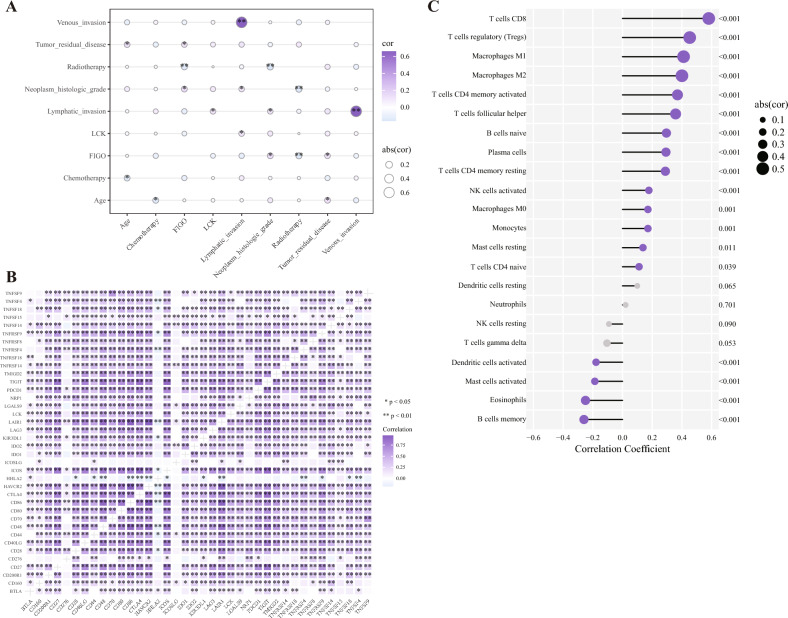
Spearman correlation analysis of the correlation of LCK with tumor clinical features, immune genes, and immune cell infiltration. (**A**) Circle map between LCK and tumor clinical features. (**B**) Correlation heat map between LCK and immune genes. (**C**) Lollipop chart between LCK and immune cell infiltration. *p < 0.05; **p < 0.01; ***p < 0.001; *LCK* lymphocyte cell-specific protein-tyrosine kinase, *FIGO* international federation of the gynecology and obstetrics, *TNFSF* tumor necrosis factor superfamily member, *TNFRSF* tumor necrosis factor receptor superfamily member, *TNFRSF* tumor necrosis factor receptor superfamily member, *TMIGD2* transmembrane and immunoglobulin domain containing 2, *TIGIT* T cell immunoreceptor with Ig and ITIM domains, *PDCD1* programmed cell death 1, *NRP1* neuropilin 1, *LAIR1* leukocyte-associated immunoglobulin-like receptor 1, *LAG3* lymphocyte-activation gene 3, *KIR3DL1* killer Ig-like receptor 3DL1, *IDO* indoleamine 2,3-dioxygenase, *ICOSLG* inducible T cell costimulator ligand, *ICOS* inducible T cell costimulatory, *HHLA* Hamburger Hafen und Logistik AG, *HAVCR2* hepatitis A virus cellular receptor 2, *CTLA4* cytotoxic T lymphocyte antigen 4, *CD* cluster of differentiation, *BTLA* B and T lymphocyte attenuator, *NK* natural killer cells.

LCK was associated with lymph node invasion (*p* < 0.05), and radiotherapy was positively associated with FIGO stage (*p* < 0.01) (Fig. 5A). LCK expression was also strongly correlated with that of immune genes, such as CD27, CD48, and CD28 (*p* < 0.01) (Fig. 5B) as well as infiltration of T cells CD8 (*p* < 0.01) (Fig. 5C). There was a significant negative relationship between LCK expression and memory B cells (*p* < 0.01) (Fig. 5C).

#### Assessment of differentially expressed genes between high- and low-LCK-expression groups

**Figure 6:**
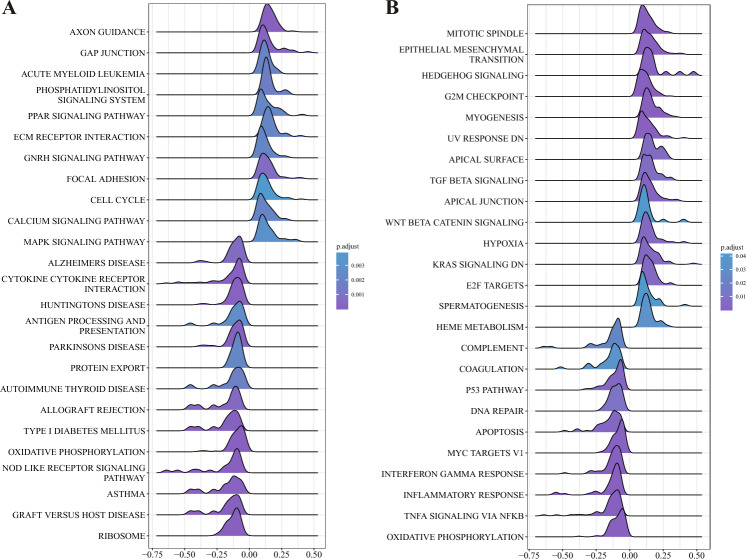
Enrichment analysis results of GSEA in the KEGG gene set and the Hallmark gene set. (**A**) Mountain map of the top 25 most enriched pathways in the KEGG gene set. (**B**) Mountain map of the top 25 most enriched pathways in the Hallmark gene set.

The top 25 most enriched pathways in the KEGG gene set were visualized by GSEA, as shown in Fig. 6A. The differentially expressed genes in the low-LCK-expression group were significantly enriched in the NOD-like receptor signaling pathway, whereas those of the high-LCK-expression group were significantly enriched in the peroxisome proliferator-activated receptor signaling pathway. GSEA similarly visualized the top 25 most enriched pathways for the Hallmark gene set, as depicted in Fig. 6B. The differentially expressed genes in the low-LCK-expression group were highly enriched in the p53 pathway and in DNA repair. In contrast, those of the high-LCK-expression group were highly enriched in E2F targets (genes involved in DNA replication) and the G2/M damage checkpoint (which prevents cells from entering mitosis when DNA is damaged).

### Construction and assessment of the multimodal radiomic machine learning model

#### Radiomic signature extraction/selection and model construction

The median ICC value for the radiomic signature was 0.947. There were 84 radiomic features with an ICC ≥ 0.75 (78.5% of all features), which were entered into the subsequent screening. After feature reduction by recursive feature elimination, six features were chosen to create the radiomic signature for the prognostic model, comprising gray level run length matrix (glrlm)_RunEntropy, gray level size zone matrix (glszm)_GrayLevelNonUniformityNormalized, glszm_GrayLevelVariance, glszm_SmallAreaHighGrayLevelEmphasis, gray-level cooccurrence matrix (glcm)_Correlation, and firstorder_90Percentile. Their important were 0.729, 0.519, 0.531, 0.577, 0.639, and 0.648, respectively. The Radscore is defined as follows,

1 $$\begin{aligned} Radscore &= 2.667 - 0.961 \times gRE - 7.716 \times gGLN - 0.495 \times gGLV \\ &\quad - 0.006 \times gSAH - 7.213 \times gC + 0.017 \times fP, \end{aligned}$$

where *gRE* is the value of glrlm_RunEntropy, *gGLN* is the value of glszm_GrayLevelNonUniformityNormalized, *gGLV* is the value of glszm_GrayLevelNonUniformityNormalized, *gSAH* is the value of glszm_SmallAreaHighGrayLevelEmphasis, *gC* is the value of glcm_Correlation, and *fP* is the value of firstorder_90Percentile.

#### Performance assessment of the radiomic model for predicting the level of LCK expression with five-fold cross-validation

**Figure 7:**
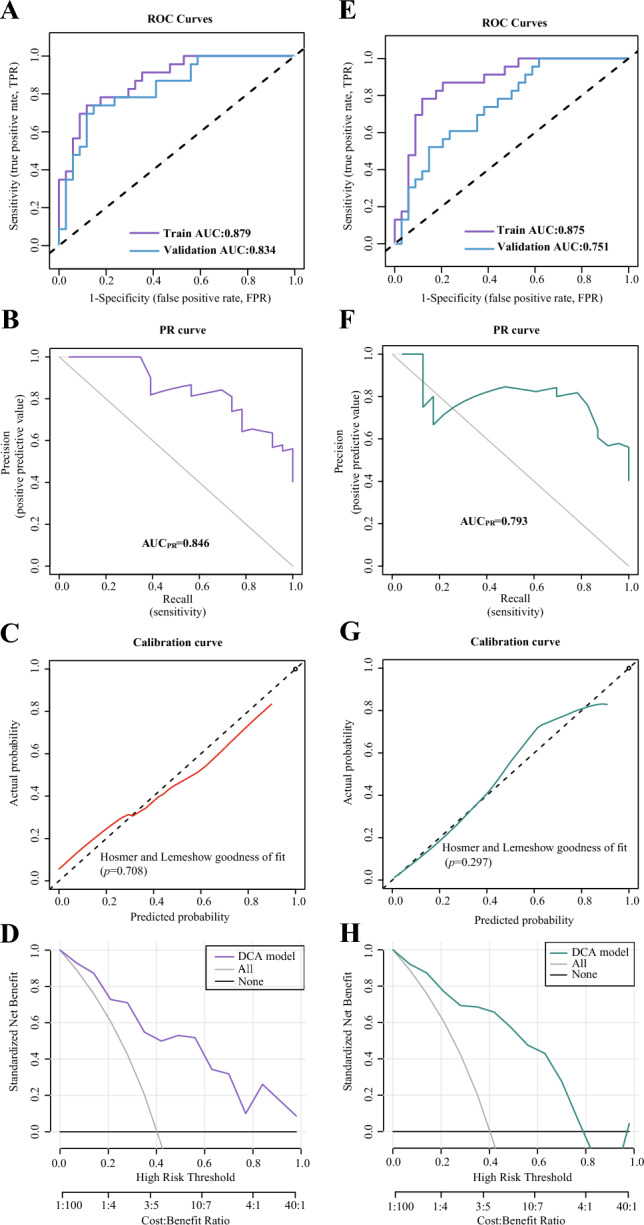
The performance of the LR and SVM radiomic models for predicting the LCK expression level during training and validation within the five-fold cross-validation. (**A**) ROC curves analysis of the LR radiomic model. (**B**) PR curve analysis of the LR radiomic model. The average accuracy, known as the PR-AUC, is computed for each coverage threshold. A more pronounced upper-right convex curve indicates superior model performance. (**C**) Calibration curve analysis of the LR radiomic model. A calibration curve illustrates the agreement between predicted and actual LCK expression levels. The ideal prediction performance is depicted by the dotted black line at a 45-degree angle, while the solid red line represents the LR radiomic model's performance. The closer the solid red line aligns with the dotted line, the higher the accuracy of the model's predictions. (**D**) DCA analysis of the LR radiomic model. The net benefit is measured on the y-axis. The purple curve represents the LR radiomic model, the gray curve represents the assumption that all patients received treatment, and the straight black line at the bottom of the figure symbolizes the assumption that no patients were treated. (**E**) ROC curves analysis of the SVM radiomic model. (**F**) PR curve analysis of the SVM radiomic model. (**G**) Calibration curve analysis of the SVM radiomic model. The solid green line represents the SVM radiomic model's performance. (**H**) DCA analysis of the SVM radiomic model. The green curve represents the SVM radiomic model, the gray curve represents the assumption that all patients received treatment, and the straight black line at the bottom of the figure symbolizes the assumption that no patients were treated.

Both the LR and SVM radiomic models were generated using the Radscore. In the training set of LR radiomic model, the accuracy, sensitivity, specificity, PPV, NPV, and Brier score were 0.825, 0.739, 0.882, 0.81, 0.833, and 0.139, respectively. In the validation set, the accuracy, sensitivity, specificity, PPV, NPV, and Brier score were 0.807, 0.739, 0.853, 0.773, 0.829, and 0.169, respectively. As shown in the ROC plot (Fig. 7A), the LR model had an AUC value of 0.879 (95% CI 0.791–0.966) in the training set and 0.834 (95% CI 0.727–0.94) in the validation set. The AUC value of the PR curve reached 0.846 (Fig. 7B). The calibration curve and Hosmer–Lemeshow test revealed excellent agreement between the predicted values of the LR model and the actual values in terms of whether or not the LCK gene was highly expressed (*p* = 0.708) (Fig. 7C). The DCA revealed that the model had a high clinical utility (Fig. 7D). Furthermore, according to the Delong test, AUC values during training and validation within the five-fold cross-validation did not statistically differ (*p* = 0.521). Therefore, the LR radiomic model exhibited a good prediction performance.

In the training set of SVM radiomic model, the accuracy, sensitivity, specificity, PPV, NPV, and Brier score were 0.842, 0.783, 0.882, 0.818, 0.857, and 0.157, respectively. In the validation set, the accuracy, sensitivity, specificity, PPV, NPV, and Brier score were 0.632, 1.000, 0.382, 0.523, 1.000, and 0.196, respectively. As shown in the ROC plot (Fig. 7E), the SVM model had an AUC value of 0.875 (95% CI 0.782–0.968) in the training set and 0.751 (95% CI 0.625–0.876) in the validation set. The AUC values of the PR curve reached 0.793 (Fig. 7F). The calibration curve and Hosmer–Lemeshow test revealed excellent agreement between the predicted values of the SVM model and the actual values in terms of whether or not the LCK gene was highly expressed (*p* = 0.297) (Fig. 7G). Again, the DCA confirmed that this model had a high clinical utility (Fig. 7H). AUC values during training and validation within the five-fold cross-validation did not statistically differ, according to the Delong test (*p* = 0.365). Therefore, the SVM radiomic model also exhibited a good prediction performance.

**Figure 8:**
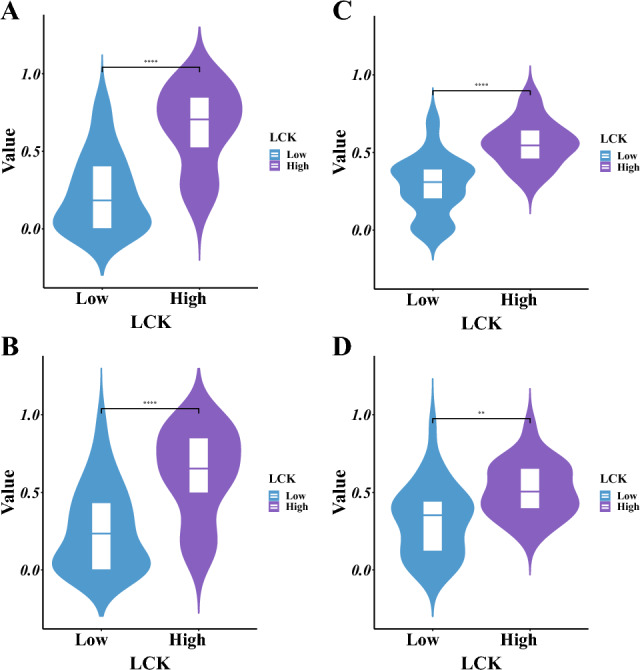
Differences in the predicted probabilities of the LR radiomic model and SVM radiomic model between the high and low LCK expression groups. (**A**) Violin plots of predicted probabilities of the LR radiomic model between the high and low LCK expression groups in the training set. (**B**) Violin plots of predicted probabilities of the LR radiomic model between the high and low LCK expression groups in the validation set. (**C**) Violin plots of predicted probabilities of the SVM radiomic model between the high and low LCK expression groups in the training set. (**D**) Violin plots of predicted probabilities of the SVM radiomic model between the high and low LCK expression groups in the validation set. *p < 0.05; **p < 0.01; ***p < 0.001; ****p < 0.0001.

The individual differences between the predicted values of the LR and SVM radiomic models for high- and low-LCK expression are displayed in Fig. 8. In both the training and validation sets within the five-fold cross-validation, there were significant differences in the radiomic score distribution between the high- and low-LCK-expression groups. The radiomic score was higher in the high-LCK-expression group.

As already described, Delong tests indicated no statistical difference in the AUC values during training and validation within the five-fold cross-validation for either the LR or SVM radiomic model. However, the Hosmer–Lemeshow test and the PR-AUC showed that the LR radiomic model somewhat outperformed the SVM model when the AUC values of the training and validation sets were combined. Therefore, the subsequent prognostic analysis was performed using the Radscore-predicted value of the LR radiomic model.

#### Performance assessment of the multimodal radiomic model

**Figure 9:**
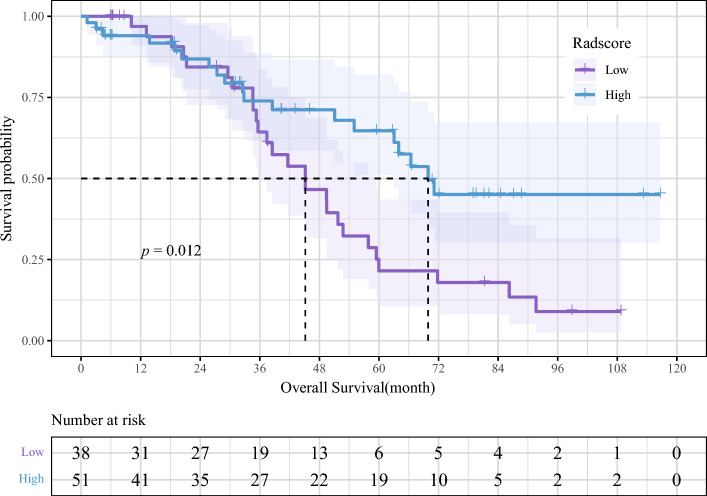
Correlations between Radscore and OS in patients with HGSOC.

Using high and low Radscore as a grouping criterium, a baseline radiomic and clinical data table was constructed (Table 2). There was no statistically significant difference in each clinical variable (*p* > 0.05). The mean survival time of patients with HGSOC exhibiting a low Radscore was 46 months, and that of patients with a high Radscore was 70 months. A high Radscore was substantially related to a better OS according to the K–M curve (*p* = 0.012) (Fig. 9).

**Table 2:** Clinical characteristics of patients with HGSOC by high and low radiomic score group from TCIA-TCGA data.

Characteristic	Total (N = 89) No. (%)	High (N = 51) No. (%)	Low (N = 38) No. (%)	*p*
Age				0.141
< 59	42 (47.2)	28 (54.9)	14 (36.8)	
≥ 60	47 (52.8)	23 (45.1)	24 (63.2)	
Radiotherapy				1
No	86 (96.6)	49 (96.1)	37 (97.4)	
Yes	3 (3.4)	2 (3.9)	1 (2.6)	
Chemotherapy				0.133
No	4 (4.5)	4 (7.8)	0 (0)	
Yes	85 (95.5)	47 (92.2)	38 (100)	
Lymphatic invasion				0.411
No/unknown	66 (74.2)	40 (78.4)	26 (68.4)	
Yes	23 (25.8)	11 (21.6)	12 (31.6)	
Venous invasion				0.853
No/unknown	73 (82)	41 (80.4)	32 (84.2)	
Yes	16 (18)	10 (19.6)	6 (15.8)	
FIGO stage				0.296
II/III	47 (52.8)	24 (47.1)	23 (60.5)	
IV/unknown	42 (47.2)	27 (52.9)	15 (39.5)	
Tumor residual disease				0.063
No/unknown	32 (36)	23 (45.1)	9 (23.7)	
Yes	57 (64)	28 (54.9)	29 (76.3)	
Histologic grade				0.488
G1/G2	9 (10.1)	4 (7.8)	5 (13.2)	
G3/GX	80 (89.9)	47 (92.2)	33 (86.8)

### Prognostic significance of the multimodal radiomic nomogram

**Figure 10:**
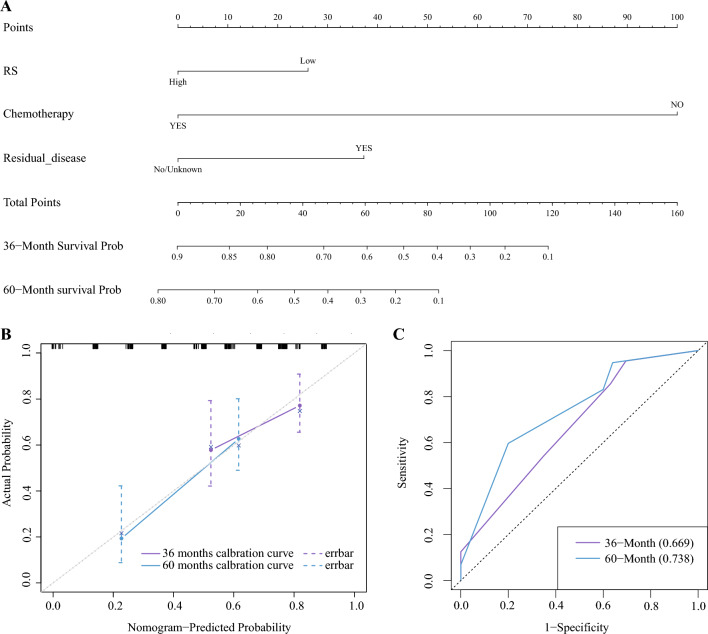
Nomogram and model evaluation. (**A**) A radiomic nomogram combining the radiomic characteristic from computed tomography images to predict the OS of a patient with HGSOC. (**B**) Calibration curve of the radiomic nomogram. The blue and purple solid lines show how the nomogram performed, while the diagonal dotted line represents an ideal evaluation. A better evaluation is indicated by a closer fit to the dotted diagonal line. (**C**) The time-dependent ROC curves analysis.

We developed a radiomic nomogram that combined clinical information with the signature from the radiomic score (Fig. 10A). This nomogram showed good calibration, as depicted in Fig. 10B. In the calibration plot, the curves at each time point are located near the diagonal line, indicating that the prediction error is small. DCA at 60 months demonstrated the high clinical utility of our model within thresholds of 0.25 and 0.7. Furthermore, time-independent ROC analysis confirmed that the radiomic nomogram had an excellent prognostic value (Fig. 10C). The AUC value of the model’s predictive power for patient OS at 60 months was 0.738.

## Discussion

Over 75% of women diagnosed with advanced OC have an alarming five-year survival rate of only 15–25%^43^. Patients with HGSOC also face poor prognoses and outcomes^5^, making accurate predictions essential for effective treatment. While various factors have been associated with HGSOC prognosis, such as patient age, pathological stage, tumor recurrence after debulking surgery, and genomic information^44^. However, these factors do not explain the heterogeneity of clinical outcomes. Tumor heterogeneity in HGSOC is linked to undesirable clinical effects. Radiomics, a quantitative analysis of tumor heterogeneity using radiographic images, can provide valuable insights. In this study, we applied a novel CT-based multimodal radiomic approach to predict the expression of LCK, a key immune-related molecule, and assess its relationship with clinical prognosis in patients with HGSOC. Our results, primarily based on patients with advanced HGSOC, revealed significant associations between radiomic characteristics, LCK expression levels, and OS in patients with HGSOC. To the best of our knowledge, this is the first study to utilize CT-based multimodal radiomics for non-invasive prediction of LCK expression and HGSOC prognosis, opening avenues for personalized clinical decision-making and treatment advancements. Our findings can be summarized as follows: (1) A high expression of LCK was significantly associated with improved OS, as demonstrated by the K–M curve (*p* = 0.042). (2) We developed a predictive model based on six radiomic features, which exhibited AUCs of 0.879 (95% CI 0.791–0.966) in the training set and 0.834 (95% CI 0.727–0.94) in the validation set according to the ROC curve. (3) Patients with a high Radsocre had better OS compared to those with a low Radsocre (*p* = 0.012).

Recently, there has been increasing interest in the potential application of immune therapy in OC. Key among the molecules involved in the immune response is LCK, which not only serves as a crucial component of the immune system, but also acts as a prognostic biomarker that regulates the TME^11^. Numerous studies have demonstrated a significant correlation between LCK expression and the prognosis of OC patients^13, 45^. Crean-Tate et al. Found a positive association between increased LCK expression and poorer clinical outcomes in endometrioid OC^46^. LCK has been extensively studied in various cancer types as well as normal tissues^47^. LCK signaling pathway has been implicated in intraocular immunopathogenesis^48^. Therefore, our study focused on the differential analysis of the immune-related components in the tumor microenvironment, and explored their associations with LCK expression, survival outcomes, tumor characteristics, and immune cell infiltration in patients with HGSOC. Additionally, we conducted an enrichment analysis of differentially expressed genes between patient groups with high and low LCK expression. High expression of LCK was linked to a better prognosis in HGSOC patients, according to the results of multiplex Cox regression analyses. Furthermore, lymphatic invasion—a clinical tumor characteristic—of immune genes, including CD27, CD48, and CD28, as well as infiltration of CD8^+^ T cells exhibited a positive and substantial connection with LCK expression (in terms of immune cells). Significantly lower LCK levels were inversely correlated with memory B cells. However, the invasive nature of LCK detection and the lack of non-invasive techniques for predicting LCK levels in OC pose significant challenges. Considering the importance of LCK as an immune system molecule, our study sheds light on its link to HGSOC prognosis. Consequently, we focused on the examination of immune-related elements in the tumor microenvironment.

Accurate survival predictions are crucial for optimal medical decision-making, particularly in the treatment of malignant tumors like HGSOC. Several techniques have been employed for survival prediction in HGSOC patients, but histological sampling and genetic analysis have limitations. Radiomics, which captures disease heterogeneity, has emerged as an optimal method^49^. Through the extraction of quantitative information from medical images, radiomics coupled with machine learning has become a precise tool in clinical diagnostics and treatment^50–53^. Chen et al. successfully differentiated between high- and low-risk HGSOC patients using a radiomic nomogram^24^. Rizzo Stefania et al. demonstrated the significant association between radiomic characteristics and prognostic factors such as residual tumors at surgical procedures and disease progression within a year in OC patients^54^. In our study, we analyzed the prognostic performance of radiomics for assessing HGSOC and its correlation with LCK expression to evaluate the utility of radiomic features in capturing phenotypic variations of ovarian tumors. By conducting recursive feature elimination screening, we identified six highly correlated radiomic features (glrlm_RunEntropy, glszm_GrayLevelNonUniformityNormalized, glszm_GrayLevelVariance, glszm_SmallAreaHighGrayLevelEmphasis, glcm_Correlation, and firstorder_90Percentile) that are highly correlated with the prognosis of HGSOC. Poor prognosis tumors exhibited characteristics such as larger tumor volume, infiltrative margins, and higher heterogeneity^7, 55^. The glszm feature describes the number of connected voxels in an image with the same gray intensity, while the glcm feature represents the joint probability of specific pixel sets with specific gray values^56^. The glcm feature demonstrates greater robustness to imaging parameters compared to other features in the original images^57^. Both glszm and glcm features are commonly used in HGSOC research for image-based classification as they capture texture and statistical correlations between pixels, aiding in the characterization of tumor heterogeneity^58^. Texture parameters are gaining increased attention from researchers due to their potential in diagnosis, treatment prediction, and prognosis assessment^59, 60^. Previous studies in ovarian cancer have shown predictive value for tumor prognosis and differentiation using glszm and glcm features^24, 61^. The repeatable and non-invasive nature of radiomics makes them potentially applicable in routine clinical practice.

To address the current lack of non-invasive methods for predicting LCK levels in HGSOC, we developed an imaging-based radiomic model for non-invasive LCK prediction. Our study found that the LR radiomic model outperformed the SVM radiomic model when assessing LCK expression in patients with HGSOC. Combining radiomic features with clinical characteristics significantly improved the LR model's prediction compared to using radiomic features alone. Moreover, our DCA at 60 months demonstrated that a high expression of LCK was associated with a better survival rate in patients with HGSOC (*p* < 0.05), demonstrating that a high Radscore indicated a higher rate of OS. We deduced that non-invasive LCK expression prediction using radiomics was beneficial for clinical decision-making. In our study, we built a multimodal model incorporating bioinformatics, radiomics, and clinical characteristics, enabling the prognostic assessment of HGSOC patients. This CT-based multimodal radiomic model served as a non-invasive method to predict LCK expression and OS in patients with HGSOC, representing a practical means of enhancing clinical prognosis.

The application of machine learning in cancer classification and prediction is expanding and has tremendous potential. In this study, we demonstrated the improved predictive accuracy achieved by combining multiscale biomedical imaging, clinical information, and genomic data. Our results highlight that radiomic features extracted from medical imaging capture distinct phenotypic variations in HGSOC, and validate the prognostic power of the developed radiomic model. Therefore, this model holds promise for translation into the clinical setting, offering a valuable tool for prognostic assessment of HGSOC.

## Conclusion

LCK plays a crucial role as a prognostic marker in HGSOC. Conventionally, tissue samples must be sequenced or immunohistochemically analyzed to determine LCK expression. However, our study reveals that radiomics can effectively predict LCK expression and prognosis noninvasively, offering significant clinical value. Our model, developed primarily using advanced HGSOC patients, demonstrates strong predictive efficacy for LCK expression and prognosis. By utilizing radiomics instead of direct LCK measurements, we found that radiomic results align with prognosis, further validating the technique's validity. Radiomics can capture the heterogeneity of HGSOC and thereby offer a way to formulate an individualized treatment plan. Combining radiomics with clinical and genetic information, our study presents a promising approach to enhancing HGSOC prognosis prediction.

## Data Availability

The datasets analysed during the current study are available form The Cancer Imaging Archive (TCIA, https://www.cancerimagingarchive.net/), The Cancer Genome Atlas (TCGA, https://portal.gdc.cancer.gov/), The Genotype–Tissue Expression dataset (GTEx, https://gtexportal.org/home/) and The University of California Santa Cruz Xena (https://xenabrowser.net/datapages/).
